# Cognition in Healthy Aging

**DOI:** 10.3390/ijerph18030962

**Published:** 2021-01-22

**Authors:** Macarena Sánchez-Izquierdo, Rocío Fernández-Ballesteros

**Affiliations:** 1 Department of Psychology, Universidad Pontificia Comillas, 28049 Madrid, Spain; 2 Department of Psychobiology and Health, Autonomous University of Madrid, 28049 Madrid, Spain; r.fballesteros@uam.es

**Keywords:** cognitive aging, healthy cognitive aging, cognitive change, cognitive trajectories, intelligence across life span, well being

## Abstract

The study of cognitive change across a life span, both in pathological and healthy samples, has been heavily influenced by developments in cognitive psychology as a theoretical paradigm, neuropsychology and other bio-medical fields; this alongside the increase in new longitudinal and cohort designs, complemented in the last decades by the evaluation of experimental interventions. Here, a review of aging databases was conducted, looking for the most relevant studies carried out on cognitive functioning in healthy older adults. The aim was to review not only longitudinal, cross-sectional or cohort studies, but also by intervention program evaluations. The most important studies, searching for long-term patterns of stability and change of cognitive measures across a life span and in old age, have shown a great range of inter-individual variability in cognitive functioning changes attributed to age. Furthermore, intellectual functioning in healthy individuals seems to decline rather late in life, if ever, as shown in longitudinal studies where age-related decline of cognitive functioning occurs later in life than indicated by cross-sectional studies. The longitudinal evidence and experimental trials have shown the benefits of aerobic physical exercise and an intellectually engaged lifestyle, suggesting that bio-psycho-socioenvironmental factors concurrently with age predict or determine both positive or negative change or stability in cognition in later life.

## 1. Introduction

### 1.1. Historical Antecedents

**Figure 1:**
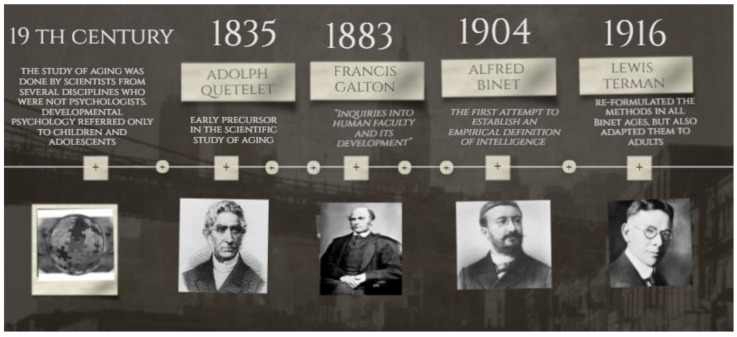
Main historical antecedents in the study of aging.

When psychology was born as a science (see Figure 1) in the last third of the 19th century, life expectancy was less than 40 years. The combination of an increasing life expectancy (due to decreasing mortality) and the reduction in fertility determined changes in the population range of children and older adults (in industrializing countries, those over 60 made up less than 5%, while there were three times more people younger than 14). Therefore, developmental psychology initially referred only to children and adolescents, with most of the early work in the study of aging being done by scientists from several disciplines who were not psychologists.

Authors agree that an early pioneer in the scientific study of aging in the 19th century was the Belgian statistician and astronomer Adolphe Quêtelet, who said: “man is born, grows up, and dies, according to certain laws which have never been properly investigated, either as a whole or in the mode of their mutual reactions”; thus, some “proper investigations” about changes along aging will be described here [1] (p. 660).

Perhaps the first empirical researcher on aging was the British human geneticist Francis Galton (1822–1911), who in 1883 published “Inquiries into human faculty and its development”, devoted to the analysis of a set of physical and psychological functions from sensitivity to mental imagery. This essay was the background for establishing his Anthropometric. About nine thousand individuals (men and women from age 5 to 80) were assessed. Galton at this time was already suggesting the importance of longitudinal studies, arguing not only that in cross-sectional studies age differences are confounded with cohort differences but also that inter-individual differences in intra-individual change can only be identified with such longitudinal designs (see [2]).

Nevertheless, as Schaie emphasized, the Mental Testing Movement—already established by psychologists—could be considered the core of the study of cognitive or intellectual competences, with individual differences attributed to age as well as along a life course [3]. This movement started with the first attempt to establish an empirical definition of intelligence under the efforts of Binet [4] and Binet and Simon [5]. Since these methods were developed to be administered to children, however, a continuation of Binet’s works was necessary. This was performed by the North American Lewis Terman, who not only re-formulated the methods in all Binet ages, but also adapted them to adults [6].

The most important aspect of this Mental Test Movement was, however, that it opened the windows to the study of intelligence and cognitive abilities, aptitudes and competences not only in children and adolescents, but throughout a life cycle. It must be emphasized that at the same time, at the very beginning of the 20th century, life expectancy in developed countries was slowly beginning to grow, and initial projections of population aging highlighted the importance of the study of aging. Therefore, as August Comte stated (“savoir, pour prévoir, afin de pouvoir”, or “knowing, to foresee, in order to be able to”), knowledge about changes in cognition across a life span started being crucial for improving the aging process, extending life expectancy and disability-free life expectancy, as well as for changing negative aging stereotypes, prejudices and discrimination, changing social policies, empowering an aging society and having an impact on successful longevity and wellbeing.

### 1.2. Methodological Issues

Taking into consideration the two methods of scientific psychology: experimental and correlational [7], the study of cognitive change attributed to age is mainly observational/correlational, for the simple reason that the underlying hypothesis is that age is the independent variable exerting a causal role on cognitive functioning and cannot be experimentally manipulated. Methodologists usually consider these methods as prospective ex-post-facto designs based on a “manipulation” of age (cross-sectional) or of time/cohort (longitudinal/sequential) [7,8,9]. Nevertheless, experimental and quasi-experimental methods are administered when researchers want to verify the determinants of those changes or intervene in those cognitive changes.

As Rabbit pointed out, the primary assumption for studying cognitive change across a life span do not depend on time or age but “is that individuals’ trajectories of change are determined by complex interactions between a great variety of factors including genetic inheritance, uterine and infant environments, levels of economic advantage and lifestyle, exposure to diseases, toxicity and stress, and access to health education and medical aid” [10] (p. 190). In the same theoretical position, the socio-cognitive theory by Bandura [11,12] posited the transactions among the person (organism), his/her behavior and the environment at the micro (the individual), meso (the family) and macro (global context) levels, as proposed by Bronfenbrenner [13]. All of these transactions act throughout a life span. It must be emphasized from the very beginning of this manuscript that, given the extreme difficulty in the study of cognitive change, there is not a single best method to examine such as complex scientific subject.

As already mentioned, the study of cognitive change attributed to age and across a life span is performed through ex-post-facto prospective designs (see cross-sectional and longitudinal). Moreover, in both cases, the observation of cognitive change is collected across specific techniques (intelligence tests, cognitive tasks, physiological measures, neurological images, etc.), or even through experimental tasks, such as a cognitive plasticity examination. Finally, data can be examined using a variety of statistical methods for data analysis, depending on the study objectives and the hypotheses formulated.

In cross-sectional studies, considered a static approach, several age groups are examined with the same assessment instruments at a specific point in time, and statistical analyses are performed between groups as basic tools for testing age group differences, or inter-individual differences. As Schaie emphasizes, “cross-sectional data representing age differences can model change over time only in the case of a perfectly stable environment and in the absence of cohort differences” [3] (p. 4). Since this assertion could be considered mistaken, cross-sectional studies—as Galton already assumed—confound age effects with socio-historical and environmental changes. Nevertheless, when the research aim is to assess inter-individual differences at a certain time (e.g., for selection purposes), this design could be efficient because it can yield age profiles related to the key targets assessed. This would, however, not be the case for basic aging research when not only age must be taken as a causal variable, because, as scholars recognize, time has no causal variables and/or covariates providing mechanisms of change [3]. In sum, in this paper, cross-sectional studies are described, providing age profiles based on age differences yielded at a particular point in time. Thus, the selection of a representative sample by age and other relevant circumstances to age change (such as education, socio-economic status, gender, etc.) and selection of the most appropriate measurement instruments to show the psychometric properties of the population involved are the most important characteristics for data quality. It must nevertheless be taken into consideration that although these studies may be efficient and convenient for practical purposes, given the time required for measurement, the results cannot be generalized to age changes or to cohort differences, and it can also be assumed that results yielded by cross-sectional studies usually maximize age differences.

Longitudinal designs are considered to be a dynamic approach to the study of aging; here, a sample of individuals of the same age is repeatedly tested across their life spans, thus providing information about their trajectories, or long-term patterns of stability and change in bio-psycho-social characteristics, as well as possibly registering the occurrence of transitions or life events. The simplest longitudinal design is that in which a cohort, i.e., a group with the same age, is followed across a long period of time and assessed at specific time intervals (e.g., every five years). Longitudinal studies also could be sequential or cohort designs; thus, it is possible to make comparisons between groups of individuals with the same age belonging to different cohorts (born at different times) tested at different times of measurement [14,15].

Longitudinal studies have different formats: those designed for the study of childhood development; those covering the entire adulthood and beyond; and, finally, those starting in later life (e.g., at age 50, 60 or beyond) or even the very old (nonagenarian, centenarian). This paper attempts to reveal what happens with cognition along a life span. Studies covering childhood to old age have thus been taken into consideration, alongside studies focused on old people—those over 60 years old—with the aim of studying healthy aging trajectories in later life.

Thus, our main objective here is to understand the process of change across aging, based on longitudinal designs. Schaie states that longitudinal designs can provide information about the five main sources of data: intra-individual change, inter-individual variability in this intra-individual change, covariations among intra-individual change variables as well as examination of the potential causal variables of the intra-individual change and its variability [16]. However, as Galton already pointed out, and as expanded by Schaie, cross-sectional and longitudinal methods are threatened by three main design components: age, cohort and time of measurement [15]. A broader perspective was suggested by Baltes, who, after rigorous analysis of both methods, exhibited five methodological shortcomings: selective sampling, selective survival, selective drop-out, testing effects and generation effects, which can be reduced to the process of selecting and maintaining sampling (selective sampling, selective survival and selective drop-out, including generation effects) and the quality of the assessment instruments, and how the effects of learning on the instruments change across time [14].

Among these limitations of longitudinal designs, the conditions referring to attrition seem to be one of the most important threats for longitudinal studies on aging, due mainly not only to mortality but also to drop-out or refusal to participate. An example can be found in our “90+ Project”, which reported on those individuals who were assessed at the baseline of the 90+ project but who have since died, dropped out or were re-examined in the follow-up [17]. They were assessed through the European Survey on Aging Protocol (ESAP) by collecting anthropometric, health and lifestyles, bio-behavioral, psychological (including cognition, personality, emotions and motivation) and social data. After 6–14 months from the baseline, 55% individuals were re-assessed, 11% died and 34% dropped out, resulting in a 45% attrition rate. When a multidimensional indicator of “successful aging” was calculated on the baseline, 90% of those individuals who died were identified at the baseline as non-successful agers, while more than a half of those who participated were identified as successful agers. It can be concluded that among such independent but very old people, mortality is less important than participation, with contextual, behavioral and psychological factors also being relevant for distinguishing mortality, survival and participation.

Finally, a methodological issue requiring consideration is the assessment of cognitive change; in other words, what the most frequent cognitive functions assessed are and which measurement devices or instruments are administered to observe these functions.

#### 1.2.1. Cognitive Function Changes

The most frequent cognitive functions and/or mental abilities assessed, both in cross-sectional and longitudinal studies, are the following: (1) perceptual speed, measured by the accuracy of the digit–symbol substitution, digit–letter and identical picture; (b) memory, measured by activity recall, memory for text and activity recall; (c) reasoning, measured by tests of figural analogies, letter series and practical problems; (d) verbal knowledge, measured by the practical knowledge, spot-a-word and vocabulary tests; and (e) verbal fluency, measured by the tests of categories (naming names of animals), and words beginning with “s”. Those cognitive functions are considered as indicators of fluid intelligence (gf), and the last two define crystallized intelligence (gc). Other functions assessed with psychometric and neuropsychological tests are inductive reasoning, executive functioning, visuospatial ability and short-term working and episodic memory. It must be taken into consideration that we are not dealing with other conditions, such as wisdom and everyday competence, since these embrace other psychological factors and the research measurement instruments are not usually standard.

#### 1.2.2. Instruments Used

In order to assess these cognitive functions, the most frequently used general instruments or measurement devices for healthy individuals are the following: (1) mental/cognitive testing, for example, the Wechsler Adults Intelligence Tests [18], Wechsler Memory Scales [19], the Primary Mental Aptitudes tests [20,21]; (2) neuropsychological tests, for example, the Trail Making Test A (assessing attention/psychomotor speed) and Trail Making Test B (assessing executive function) [22]; (3) mental test examinations developed on the basis of medical screening for classifying mental impairment, based on categorical or nominal scaling, such as Mental State Examinations [23], which would be methodologically non-appropriate to transform into “trajectories” but which are present in some cross-sectional and longitudinal studies; and (4) cognitive experimental tasks assessing specific functioning, such as cognitive plasticity [24,25] or experimental tasks administered in outpatient situations, which allow assessment of cognitive processes within person processes [26].

The developments emerging in recent decades from the cognitive neuroscience of aging and the current sophisticated technology complementing our mental and neuropsychological tests must be taken into consideration in an attempt to understand the neurobiological aspects of aging changes and to distinguish between normal and pathological cognitive aging. In this manuscript, studies from a cognitive neuroscience perspective are introduced.

### 1.3. Healthy Aging in Later Life

As this review deals with cognitive trajectories in later life, specifically in healthy individuals, it is necessary to clarify what is understood by “healthy aging”. Rowe and Kahn (1987), alongside the usual and successful ways of aging, in their classification also consider a pathological age; thus from a biomedical perspective, healthy means the opposite of pathological, and we must therefore distinguish between pathological, usual and successful aging [27,28]. The distinction can also proceed from changes in cognition already studied, which theoretically occur from the development to the involutional stage, conceptualized as normative versus non-normative cognitive aging. Following Steinerman, however, it must be emphasized that “normative and non-normative cognitive aging are stunningly complex phenomena influenced by a broad range of factors acting on various timescales. Normative aging is associated with improving cognitive abilities through early adulthood followed by a period of relative stability during mid-life and late-life decline. Non-normative influences produce additional effects superimposed on the complex normative landscape” [25] (p. 2).

Thus, we emphasize Rowe and Khan’s words: “research in aging has emphasized average age-related losses and neglected the substantial heterogeneity of older persons. Gerontologists and geriatricians have interpreted age-associated cognitive and physiological deficits as age-determined and, therefore, the role of aging per se in these losses has often been overstated” [27] (p. 143). This fact influences population aging stereotypes, attributing and generalizing cognitive pathological aging. Therefore, in this article, we focus on healthy aging, including usual and successful ways of aging, or in other words, non-pathological aging [27,28].

The next section describes cross-sectional, longitudinal and cohort studies showing cognitive profiles and trajectories in non-pathological adults older than 60. Finally, the last section will deal with the intervening factors (biological, environmental and behavioral) in cognition across a life span and in old age.

## 2. Cognitive Functioning in Healthy Older Adults

As stated above, the study of cognitive aging requires the review of cross-sectional studies measuring, at certain times, cognitive functioning in groups of individuals at different ages, yielding inter-individual differences, while longitudinal studies yield intra-individual differences. Finally, longitudinal cohort studies allow us to establish the inter-individual differences accounted for by socio-historical change. Quasi-experimental studies also allow us to examine the effects of treatments, training, interventions or life events on cognitive functioning across a life span. Therefore, a review of the aging databases was carried out (MEDLINE and PsycINFO databases; keywords: cognition, trajectories, healthy trajectories, cognitive trajectories, healthy aging trajectories, later life, elder; number of references reviewed: 296), looking for the most relevant studies carried out on cognitive functioning in healthy older adults. A summary of the selected studies on cognitive change in healthy older adults can be found in Supplementary Table S1.

### 2.1. Cross-Sectional Studies

From the very beginning of intelligence measurement, research on aging points to an age-related decline from early to late adulthood in certain cognitive abilities, as well as growth or stability in others across a life span (for example [21,29,30]).

One of the first and most influential cross-sectional studies was designed by Jones and Conrad, who investigated the negative relations between age and cognitive performance with the Army Alpha tests administered in World War One [31,32]. These researchers collected data on 1191 individuals from several communities between 10 and 60 years of age. Small age-related effects were significant in the Arithmetic, Antonym–Synonym, Disarranged Sentences and General Information tests, but more pronounced age differences occurred with the Following Directions, Common Sense, Number Series and Verbal Analogies tests. Thus, age differences were quite substantial on some of the subtests but not others.

David Wechsler developed the first Wechsler–Bellevue Scale to respond to a perceived need for an individual adult examination of intelligence that could be widely applied and useful for clinicians in making psychological diagnoses, including cognitive impairment [18]. The standardization sample consisted of 1071 adults of 10–70 years of age, 1300 adults between 20 and 64 years of age, plus an additional 475 adults from 60 to 75 or older in the revision in 1955, and 1480 adults between 20 and 74 years of age in the 1981 revision. This study revealed remarkable findings: the growth of intelligence does not finish in adolescence; various aspects of intellectual performance show different peak ages; and decrements across different subtests at older ages were not uniform. Subsequently, several studies were carried out with the WAIS tests; while some of them [29,33] hypothesized that intelligence declines between the ages of 25 and 65, others postulated that it continues to rise to the age of 50 [34,35].

**Figure 2:**
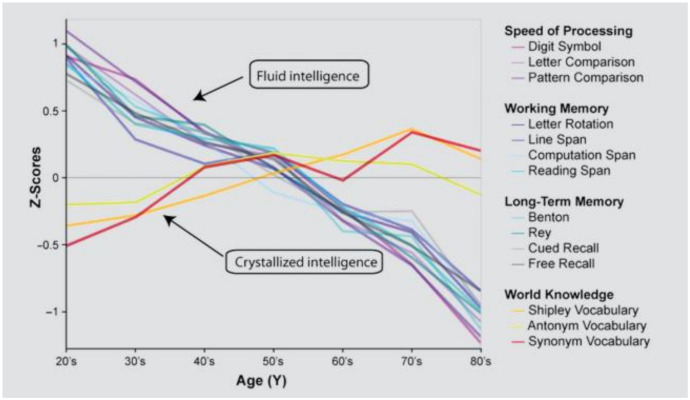
Cross-sectional aging data showing behavioral performance [40]. Cross-sectional aging data adapted from Reference [9]. showing behavioral performance on measures of speed of processing (i.e., Digit Symbol, Letter Comparison, Pattern Comparison), working memory (i.e., Letter rotation, Line span, Computation Span, Reading Span), long-term memory (i.e., Benton, Rey, Cued Recall, Free Recall), and world knowledge (i.e., Shipley Vocabulary, Antonym Vocabulary, Synonym Vocabulary). Almost all measures of cognitive function (fluid intelligence) show a decline with age, except world knowledge (crystallized intelligence), which may even show some improvement. Reproduced with permission of the publisher; from Park DC, Bischof GN. The aging mind: Neuroplasticity in response to cognitive training. Dialogues Clin Neurosci. 2013;15(1):109–119 Copyright © 2021 LLS.

Depending on the question, there are many ways to represent the domain of intelligence [36,37]. Most studies employ two types of categorization of intelligence: the fluid mechanics or the crystallized pragmatics of intelligence, initially called Intelligence A and B by Hebb [38], and the distinction proposed by Horn and Cattell between two second-order factors of psychometric intelligence, fluid and crystallized (Gf and Gc) [39]. Figure 2 presents evidence that the speed of processing, working memory, long-term memory and reasoning (Fluid intelligence) show age-related decline, even in a highly educated lifespan sample, while knowledge (crystallized intelligence) remains invariant, or even increases with age [40].

After a cross-sectional study with five age groups (*n* = 297; from 14 to 61 years), Horn and Cattell concluded that the mean level of fluid intelligence was systematically higher for younger adults (relative to older adults), while the mean level of crystallized intelligence was systematically higher for older adults (relative to younger adults) [39]. The results yielded in this study have been supported by many other studies, authors and samples (for example, [41,42,43,44]).

Thus, performance on tasks that involve working memory, processing speed and cognitive plasticity steadily declines after midlife, possibly due to an age-related loss of biological potential [45,46,47,48,49]. However, it is remarkable that cognitive skills and processes formed through cultural learning could compensate the decline in biological potential. Salthouse and colleagues have tried to answer the question about how many mechanisms contribute to the age differences in measures of cognitive functioning [43,48,49,50]. Using multivariate cross-sectional data with statistical control of variance in one variable when examining the relationship of age to other variables (reasoning, memory, speed, and vocabulary abilities), researchers demonstrated that a wide variety of cognitive and neuropsychological variables, including many measures or processes of memory, are substantially reduced with age.

Furthermore, Li et al. showed that the percentage of the predicted variance at both ends of a life span (compared with other life periods) was larger when sharing chronological age, processing speed and the two facets of intelligence (fluid and crystallized abilities), accounting for 69% of the explained variance in old age [51].

On the other hand, despite the idea of declining intelligence with increasing age and that this decline accelerates with advancing age, Park and colleagues showed through three cross-sectional studies, sampling each decade from 20 to 80 and matching younger and older adults by education, health and demographic variables and processing speed, that the magnitude of decline was as great from 20 to 30 as from 70 to 80, suggesting an equivalent loss of function across a life span. Furthermore, working memory and episodic memory did not show evidence for accelerated decline in old age. However, even though the amount of cognitive resource loss is the same for each decade, loss is accumulated through a life span and consequently greater in the later decades [40,47,52].

In terms of aging intelligence focused on old and very old individuals, the first representative study was the Berlin Aging Study (see https://www.base-berlin.mpg.de/en) [53,54,55,56]. This study was based on a representative sample of 516 older citizens aged from 70 to 103 from West Berlin. The sample was stratified by age and gender, resulting in 43 men and 43 women in each of 6 different age groups (70–74, 75–79, 80–84, 85–89, 90–94 and 95+). A battery of 15 psychometric variables was administered to participants. The five ability factors were Mental Mapping (perceptual speed), Memory, Reasoning, Verbal Knowledge and Verbal Fluency. The first three abilities were loaded in fluid intelligence, and the last two in crystallized intelligence.

Data from this study demonstrated a great “range of individual variability on a large battery of cognitive tasks and intelligence tests” [56]. Furthermore, among the very old, specifically, the 90-year-olds, there were some who functioned above the average of the 70-year-olds [53]. Thus, this study supported the notion that mechanical abilities tend to decline earlier than pragmatic abilities, specifically the negative age correlations for the three mechanical abilities (perceptual speed, memory and reasoning) were significantly higher than those for the two pragmatic abilities (knowledge and fluency). Up to the age of 70, the aging trajectories of the mechanics and the pragmatics of intelligence differ; thus, the differences between the two dimensions of intelligence appear to decrease with increasing age [56], and this distinction, compared with earlier periods of a life span, appears to be less pronounced [55].

The results also suggest the importance of the sensoriomotor functioning related to intellectual functioning, which accounted for 59% of the total variance in general intelligence [56], and “differences in intellectual functioning in old and very old age showed a greater degree of consistency (homogeneity) across abilities and ability domains than differences in intellectual functioning during earlier periods of the adult life-span”: a substantial amount of inter-individual difference was related to perceptual speed (38% of the reliable variance), while only about a third was related to chronological age [55] (p. 339).

A cross-sectional study of 338 elderly participants by Hatta et al. compared the developmental profiles of four age groups (50s, 60s, 70s and 80s) in verbal memory and visuospatial task performance [57]. Individual cognitive functions were assessed with the Nagoya University Cognitive Assessment Battery (NU-CAB) [58]. Data from the study show that performance differences in verbal memory and visuospatial tasks in young individuals decreased in the older age groups.

In conclusion, although cross-sectional comparisons reveal age-related cognitive declines beginning in their 20s [16,29,48], there is a mixture of maturational and learning influences [59,60]. As addressed by the cross-sectional authors, older adults show a great range of inter-individual variability in cognitive functioning due to age but authors agree that this decline occurs in some abilities, while there is stability or even growth in others. Thus, higher scores in crystallized intelligence and lower scores on fluid intelligence (relative to younger adults) have been systematically revealed. Finally, the authors stated that those inter-individual differences are not only explained by chronological age but the variance is also shared between processing speed and the two facets of intelligence (fluid and crystallized abilities).

### 2.2. Longitudinal Studies

In the middle of the 20th century, the first longitudinal studies on aging appeared, which included people who had reached middle adulthood (for example, [34,61,62]). These studies revealed that most abilities were maintained at least into midlife, contrasting with the results of earlier cross-sectional research [29,33,63,64,65,66,67].

The first representative longitudinal study reaching old age was the Seattle Longitudinal Study (see https://sharepoint.washington.edu/uwsom/sls/about/Pages/default.aspx) [3,16,65,66,67]. Schaie administered the PMA battery to large samples of adults from 1956 and continued over seven intervals, organized into 7-year age groups [20,21]. The recruitment procedures and inclusion criteria were similar each year. A cross-sectional sample of 4850 adults completed the five primary tests, of whom 2777 returned for a 7-year longitudinal assessment (43% attrition). The cross-sectional sample with the latent constructs consisted of 2038 adults, of whom 1257 returned for a 7-year longitudinal assessment (38% attrition) [16] (p. 38–43).

The cognitive scores on the five primary tests (series completion reasoning, spatial orientation, number arithmetic, multiple-choice vocabulary and word fluency) were reported in T-score units based on the initial assessment of the complete sample of 4850, and on the sample of 2038 for the latent constructs [16]. The SLS revealed that intellectual abilities had a negative linear relationship to age, and although aging individuals show a great variety of decline in specific intellectual abilities, the study showed consistently different patterns of decline and stability in cognition across a life span. The overall picture showed negative age effects on fluid abilities, while numerical ability (simple arithmetic calculations) and verbal ability (synonyms and recognition tests of meaning) improve until midlife and then remain stable until the age of 81. Although most people experience measurable cognitive loss by age 60, with widespread declines by age 75, it is possible to find individuals at age 81 who perform at a higher level on vocabulary tests than people at age 25 [67]. In addition, the magnitude of decrement rises with age, but increasing age mostly affects perceptual speed [3].

The results from this study have been supported by many other longitudinal studies, authors and samples that have shown that there is no uniform pattern of age-related changes across all intellectual abilities (for example, [68,69,70]), while also showing stability for measures of crystallized ability, and a significant acceleration in linear decline after age 65 for measures with a large speed component [51].

The longitudinal study of Caskie, Schaie and Willis, with data from different cohorts aged 25 to 81, drew a similar trend to the SLS study and pointed out that higher levels of verbal ability over age 60 were associated with lower rates of non-linear change over time [71]; also, the difference between change coefficients for all abilities was much greater between ages 74 and 81 than between any other ages, contrary to other studies [47,55].

The Virginia Cognitive Aging Project (VCAP) (see http://faculty.virginia.edu/cogage/) with data from over 1400 individuals from age 18 to 99, participating on at least three occasions, supports previous cross-sectional age-related declines until age 60 and either stable or positive longitudinal changes [49,72,73,74,75,76].

However, these longitudinal studies have focused on cognitive functioning along a life span, so it is possible that the study of old age might incorporate bias through inclusion criteria by including healthy old individuals, as well as individuals with cognitive impairment. In the next section, the most important studies focusing on highly select and healthy old persons are reviewed, the results of which have been inconsistent.

The Duke Longitudinal Study (DLS-I) was the first major longitudinal study of healthy older adults [77]. In this study, started in March 1955 and ended in 1976, participants were interviewed and tested every 2 years for 22 years. The sample consisted of 270 adults, aged 60–90 at baseline. To observe “normal” aging, participants were required to be functionally healthy and living independently in the community. The description of all the measures used in the psychological part of the study can be found in Siegler [78].

The second Duke Longitudinal Study (DLS-II) developed from the first study in 1966–1967 and its first subjects were tested in 1968. The aim was to double the number of subjects and to obtain data before the conventional threshold of old age, which explains why the age range of the sample was 45–70 years. Test dates were 2 years apart and the measures included those in DLS-I with additions. The data obtained showed that subjects tested longitudinally tend to show maintenance of functions up to age 71, after which decline begins. Both the cross-sectional and longitudinal results suggested a small change in memory scores over age 60, and the major portion of the observed loss was in those tasks that involved speed components. As pointed out by Palmore et al. as well as Schaie, longitudinally the younger cohort showed significantly superior performance only for immediate and delayed logical memory [16,77].

The Swedish Betula Study (see https://ki-su-arc.se/the-betula-project/) was a prospective cohort study involving a total of 3000 subjects whose ages were 35, 40, 45, 50, 55, 60, 65, 70, 75 and 80 years at baseline [79,80]. The longitudinal assessment in 1994 consisted of 875 participants (13% attrition). For episodic memory, cross-sectional data suggest declines from age 35, while longitudinal data indicate stability up to age 60. Above that age, cross-sectional and longitudinal analyses indicate approximately the same rate of age-related cognitive decline; the overall picture is an increase in performance up to middle age followed by a decrease in the older cohorts for semantic memory, although there is no age-related decline when educational level was controlled for, and no age decline for short-term memory [80].

BASE II is the longitudinal follow-up to the German Berlin Aging Study, with a sample of 206 individuals aged 70 to 100 (BASE see https://www.base2.mpg.de/en). Data were collected during 1995–1996 from 206 survivors approximately 4 years after baseline assessment (1990–1993), and showed that the availability of sensorimotor, cognitive, personality and social resources facilitated the use of strategies adapting to losses in everyday functioning within a 4-year interval [54,81,82,83].

It seems clear that rapid changes in cognitive abilities are usually signs of disease and appear unrelated to age. As has been pointed out in several studies, an individual’s cognitive trajectory can be an indicator of decline or certain clinical conditions or even time until death [84,85,86,87]. Longitudinal studies have discovered that acceleration in cognitive decline is a symptom of some pathology, and have even revealed an acceleration of cognitive decline 3–8 years before death, specifically a more rapid decline in crystallized knowledge and episodic memory [10,77,88,89,90,91,92,93]. Using longitudinal data from 288 participants without dementia in the H70 study in Sweden, in which 70-year-olds were followed until death, Thorvaldsson et al. identified the onset of terminal decline more than 6.6 years prior to death for verbal ability, 7.8 years for spatial ability and 14.8 years for perceptual speed [94]. As evidenced by Palmore et al.’s findings, nearness to death was more closely related to intellectual decline than was chronological age [77]. Berg and Berg, Nilsson and Svanborg argue that “terminal decline” is signaled when a strong decline precedes death [95,96], as has been shown in the Gothenburg study. Nevertheless, as noted by Rabbitt, in those individuals with exceptional general health, age differences have little or no measurable effect on cognitive functioning [10].

Therefore, studies based on the terminal decline paradigm show that cognitive change is related to survival. An increasing body of empirical studies suggest that cognitive abilities are a strong predictor of survival across an entire life span; these studies suggest an association between higher cognitive ability in youth and later mortality, less morbidity and overall better health [94,97,98,99]. This field of research is known as cognitive epidemiology. Deary proposed that this association may even be ascribed to a general body system integrity in which better performance on cognitive tests also reflects the vitality of other bodily systems that make individuals adapt better to their environment [100].

On the other hand, several studies have reported that a single common factor accounts for large proportions (between approximately 30% and 60%) of individual differences in age-related changes in cognitive abilities [101,102,103], suggesting that concomitant changes in multiple domains of cognitive function, for example, the g factor [104], memory factor, speed factor [105], processing speed composite and verbal memory composite [106], are core features of cognitive aging.

With advancing age, some research shows that beyond age 85 all mental abilities seem to decline for most people [107]. This phenomenon is known as dedifferentiation of cognitive functions [81,108]; that is, “a pattern of age-related increases in the correlations among measures of cognitive functions, sensory-motor functions, and general health between ages 70 and 100” [109] (p. 39). Contrary to the dedifferentiation hypothesis, Tucker-Drob and Salthouse, in their dataset of 2227 subjects aged 24–91 from seven different studies conducted at the Cognitive Aging Lab at the University of Virginia, starting in 2001, did not find evidence for systematic increases in the magnitudes of relationships among cognitive abilities [110].

These findings allow us to ask an interesting question: do people of high and low levels of general intelligence decline at the same rate? Rabbitt affirms that individuals of high, medium and low mental ability show closely similar losses in intelligence scores over time; but, the individual starting score is important. As Rabbitt remarks, a loss of 10 score points out of an original score of 150 is not the same as a 10 score points loss of someone who had a young adult score of 80 [10].

Is it possible that some functions are maintained while others decline? Tucker-Drob summarizes a number of studies that have reported positive correlations, medium to large in magnitude, and significantly different from zero, among longitudinal changes in multiple cognitive variables [111]. This means that a person who declines quickly in one cognitive domain is also likely to decline quickly in another cognitive domain.

Finally, as is emphasized by some authors, the most important bias of longitudinal studies is attrition; that is, the individuals who participate throughout the study die or drop out. For example, Fernández-Ballesteros et al., in their longitudinal study 90+ (*n* = 188 independent older than 90 years), found attrition from baseline to follow-up of 45%, but it is relevant to note that while mortality was only 11% (lower than in the 90-year-old general population) refusal to participate was 34%; thus, refusal is three time more important that mortality for attrition [17].

In conclusion, authors have found increasing levels of cognitive variability with advancing age and a substantial intra-individual variability in cognitive performance, stable over time and across cognitive domains, that can be measured independently of the systematic effects associated with materials, practice or other influences (for example, [112,113]), although it is substantially greater in individuals experiencing neurological disturbances or experiencing more severe symptoms associated with other health problems than healthy adults [114].

### 2.3. Patterns of Generational Differences

Cohort differences are the result of historical influences, such as educational opportunities, cultural and other life style factors and socioeconomic status. Results from the SLS and from the BASE studies have demonstrated the prevalence of substantial generational (cohort) differences in cognitive abilities [3,16,54,65,81,115]. Baltes and Mayer affirm that there is a very large negative difference (1.8 SD) in performance level between cohorts aged 70 and over 95 [81]. Furthermore, longitudinal data from the BALTES study specify a systematic increase in the level of performance for both abilities (fluid and crystallized), amounting to more than 1 SD across the five 7-year cohorts.

Schaie et al. reviewed generational differences in cognitive abilities (Verbal Meaning, Space, Reasoning, Number and Word Fluency) using the parent–offspring data from their family study. At comparable ages, there seems to be an increase in performance in more recent cohorts on Number and Word Fluency, whereas the younger generation performed better than their predecessors on Verbal Meaning, Space and Reasoning [116].

Caskie, Schaie and Willis designed a cohort-sequential study, using a cohort-sequential growth model from age 25 to age 81 [71]. Data revealed the influence of cohort, gender and level of education in individual variation in cognitive performance. Specifically, cohort, gender and level of education explained individual variation in the rate of decline for spatial ability, while the rate of decline in reasoning ability was predicted by both cohort and education, and verbal ability was only predicted by cohort. Furthermore, being in a later birth cohort and having a higher level of education was associated with higher levels of ability at age 67.

In contrast, comparisons by Salthouse of composite scores for five cognitive abilities (reasoning, spatial visualization, vocabulary, verbal memory and perceptual speed) in individuals tested at different ages in different years revealed that “within-cohort differences across ages were often as large as between-cohort differences across ages” [75] (p. 123); thus, as the authors pointed out, the differences in cognitive abilities were nearly the same in within-cohort age and in between-cohort comparisons.

### 2.4. Some Discrepancies Between Cross-Sectional and Longitudinal Age Trends

There are several measurement issues in the study of aging that we have to take into account (see Table 1). Firstly, cross-sectional studies are potentially influenced by cohort differences, overestimating age-related differences (for example, [69,117]). Secondly, several studies have focused on prior experience with the test or practice effects [49,69,76,118]. Thus, longitudinal comparisons are distorted because performance on a second occasion can be influenced by the first testing occasion, often showing a higher performance than would have been the case without an initial assessment; this problem can increase with shorter retest intervals. Thirdly, we have to take into account the “Flynn effect”, which refers to the rise in general intelligence scores on IQ tests over time, specifically an IQ increment of 13.8 between 1932 and 1978 (0.3 points on the IQ per year or 3 points per decade), implying “massive IQ gains on the order of 5 to 25 points in a single generation” [119] (p. 171), most clearly for fluid rather than crystallized abilities [120,121]. New generations thus tend to have higher scores on cognitive tests than people tested in prior decades, and even the later born cohort showed steeper mortality-related declines [88,122]. As remarked by Flynn “cross-sectional data, as a measure of the effects of aging on IQ, are suspect. … Cross-sectional data compare, for example, 80-year-old subjects with a group of 20-year-old subjects, with both groups being tested at the same time. This makes sense only if current 20-year-olds have the same IQ as 20-year-olds did two generations ago, that is, when today’s 80-year-olds were 20” [119] (p. 187).

Taking this into consideration, it is possible that longitudinal comparisons may be distorted by the Flynn effect, and it is therefore necessary to consider period or time-of-measurement influences [122]; but, as has been pointed out by Fernández-Ballesteros and Juan-Espinosa, intelligence gain is not a product of biological evolution, but is influenced by the transactions between biological conditions and thousands of environmental changes and experimental conditions (such as education, nutrition, socio-economic and political and sociohistorical developments) [123].

**Table 1:** Some discrepancies between cross-sectional and longitudinal studies.

	Cross-Sectional Studies	Longitudinal Studies
Design	Several age groups are examined with the same assessment devices at a specific point in time, and statistical analyses are performed between groups as basic tools for testing age group differences, or inter-individual differences	A sample of individuals with the same age is repeatedly tested across a subjects’ life span, thus providing information about their trajectories, or long-term patterns of stability and change in bio-psycho-social characteristics, as well as possibly registering the occurrence of transitions or life events.
When this kind of studies are recommended	When the research aim is to assess inter-individual differences at a certain time (e.g., for selection purposes), because it can yield age profiles related to the key targets assessed.	Longitudinal designs can provide information about five main sources of data: intra-individual change; inter-individual variability in this intra-individual change; covariations among intra-individual change variables as well as examination of potential causal variables of the intra-individual change and its variability
Limitations	Cross-sectional studies confound age effects with socio-historical and environmental changes The results cannot be generalized to age changes or to cohort differences, and it can also be assumed that results yielded by cross-sectional studies usually maximize age differences	Cross-sectional and longitudinal methods have five methodological shortcomings: selective sampling, selective survival, selective drop-out, testing effects and generation effects, which can be reduced to the process of selecting and maintaining sampling (selective sampling, selective survival, selective drop-out, including generation effects) and the quality of assessment devices, and the effects of learning on assessment devices change across time. The most important bias of longitudinal studies is attrition
Measurement issues	Cross-sectional studies are potentially influenced by cohort differences, overestimating age-related differences (for example [69,117]).	Several studies have focused on prior experience with the test or practice effects [48,69,76,114]
When does the age decrement begin to be detectable?	These studies established an earlier age for the beginning of decline (for example [16,29,48])	Longitudinal studies have shown that the onset of average decline in cognitive abilities occurs at considerably later ages [3,59,84,85] and age-related changes from age 20 to 60 tend to be small or non-existent [65,86,124].

## 3. Intervening Factors in Cognitive Functioning

Authors agree about the relative importance of genetic factors on aging accounting for 25% in comparison with environmental or behavioral factors at 75% (for example [125,126]). As is well known, intelligence also has a high heritable rate (e.g., [127]) and predicts important educational, occupational and cognitive success as well as health outcomes, better than any other trait [128]. Additionally, however, in the same fashion that occurred during the process of aging, intelligence and aging maintain their malleability [129]. This parallelism makes Vaupel et al. postulate that “polymorphisms are present, which is supported by the evidence of increases with age in the genetic component of variation in both cognitive and physical ability!” [126] (p. 895). As has already been stated, from a socio-cognitive theory perspective, cognitive functioning at a certain point in life depends on the transaction throughout the whole life span between bio-behavioral and socio-environmental synergies (for example [41,130,131]).

**Figure 3:**
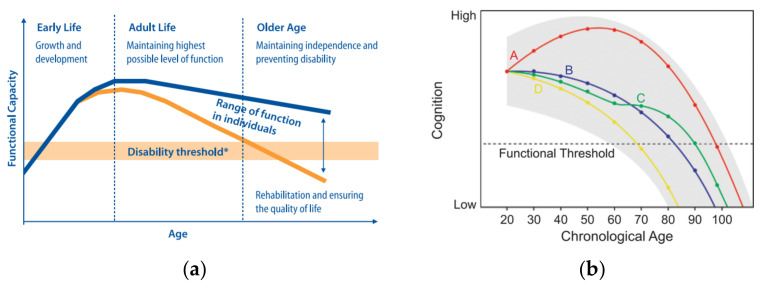
(**a**) WHO (2002) proposal about aging and functional capacities across a life cycle [56]. Reproduced with permission from WHO, Active ageing: a policy framework, Page 14;published by WHO, 2002., Copyright 2020; and (**b**) Hertzog et al.’s [57] proposal about growth and decline across a life cycle. Reproduced with permission from SAGE Publications. License 4967130282106.

Taking into consideration a set of intrinsic and extrinsic factors as determinants of aging, the WHO [132] introduces also the concept of disability threshold depending on when the individual reaches his/her maximum level during his/her processes of development and decline. In a similar way, Hertzog et al. showed different possibilities of performance depending on an individual’s intrinsic capacities or behavioral plasticity that is continuously reshaped by the individual’s environmental context, biological state, health and cognition relevant behaviors [133]. Therefore, as we can see in Figure 3, there are different possibilities of performance depending on behavioral plasticity that is continuously reshaped by the individual’s environmental context, biological state, health and cognition relevant behaviors.

The main objective of this section is to review those biological influences accounting for how cognitive abilities change along a life span (genetic and bio-medical) as well as the intervening personal psychological and behavioral factors, without intending to be exhaustive, but to give a briefly overview that can help to understand the intervening factors in cognitive functioning.

### 3.1. Bio-Medical Intervening Factors

Starting at the very beginning of the life cycle, twin studies suggest that genetic factors influence individual differences in the acceleration of cognitive decline from adulthood to old age [68]. Finkel and co-authors, with data from the Swedish Adoption/Twin Study of Aging, demonstrate a decline in late adulthood in the genetic variance of general cognitive ability, while environmental factors begin to account for more total variance in general cognitive ability in late adulthood [68,134], which replicates previous findings from other twin studies of aging [135]. Rabbitt affirms that only 13.4% of the variance in intelligence test scores between individuals can be attributed to age differences between 40 and 92 years of age [10]. This has been tested in both cross-sectional and longitudinal studies [59]. Nevertheless, as is supported by several studies, from an epigenetic perspective, some of these pathological conditions are determined by the interactions between genetics and environmental, among other, psycho-behavioral factors [129,136]. Finally, at a functional brain level, researchers suggest that declines in cognitive processes begin early in life [48,137].

Cognitive functioning seems to be associated with biological losses of the sensory systems (hearing, vision and balance) (for example [10,81,138,139,140]) and with multiple concurrent diseases or medical conditions in older adults, including cardiovascular disease, stroke, high blood pressure, hypertension or diabetes [141,142,143,144,145,146,147,148]. In terms of the effects of some of these pathologies, dementia, a neuropsychological disease, is exerting perhaps the most important role along the process of aging because of its implications in all aspects of personal and social functioning in daily life. Although there is substantial evidence that older adults show neuronal changes that can be signs of pathological aging (atrophy, plaques, Lewy bodies and vascular changes) and that these neural changes are related with other illness, it must be emphasized that they can also be present in usual aging.

Despite these pathological biomedical conditions, individual performance can still be improved in very old persons [149,150], as Park and Bischof formulate: “although there is some neural deterioration that occurs with age, the brain has the capacity to increase neural activity and develop neural scaffolding to regulate cognitive function” [40] (p. 109). It is therefore possible to improve neuronal, and thus cognitive performance, considering behavioral plasticity (the individual’s environmental context, health, and healthy behaviors), as we will see further on. Along the same lines as Park and Bischof, Fernández-Ballesteros, et al. compared a sample of healthy older adults (from 55 to 102 years old) to those with Mild Cognitive Impairment (MCI) and Alzheimer Disease patients (AD), calculating that “illness” (MCI or AD) is five times more important than “age” (healthy) in accounting for cognitive functioning variance [24].

In sum, from an epigenetic perspective, as supported by several studies, these pathological conditions are determined by the interactions between genetics and environmental factors, and among them personal behavioral repertoires learned across a life span [151], called “life styles” [136], can be considered as protective factors for healthy aging [132,152].

At this point, our concern and our major question might be: Is it possible to promote cognitive functioning? Based on the construct of “reserve capacity,” within their conceptual approach to successful aging, Baltes and Baltes posited a theoretical model called “Selective Optimization with Compensation” (SOC), postulating that older adults may be able to maintain cognitive functioning across a life span by *Selecting* all environmental conditions, which *Optimize* competences, and when needed, *Compensating* aging effects or adverse circumstances [153]. The cognitive level of performance is therefore malleable and open to enhancement throughout the human life span. This idea is supported not only by psychology and gerontology but also new research in neuroscience, supporting the new concept of neurogenesis; in other words, neural or cognitive plasticity remains in old age; new neurons as well as new synaptic connections can grow in old brains [133,154,155], and cognitive functioning can thus be improved. SOC has inspired cognitive training as well as most of the program for promoting active, healthy or successful aging over the last thirty years, with very good results (see [156].

### 3.2. Factors for Enhancing and Promoting Cognitive Functioning

Recently, the WHO defined healthy aging with arguments close to those mentioned above, by the process of developing and maintaining the functional ability that enables wellbeing in old age… depends upon their Intrinsic Capacities (IC, composite of all the physical and mental attributes…and their socio-economic and physical environments and the interaction between them” [157] (p. 2) After a deep and broad review regarding the enrichment effects on adult cognitive development, Hertzog, Kramer and Lindemberger reviewed the most important conditions (most of them psycho-behavioral) preserving and enhancing cognitive functioning in old age [133]. The course of normal aging shapes a zone of possible functioning, which reflects person-specific endowments and age-related constraints. Individuals influence whether they function in the higher or lower ranges of this zone by engaging in or refraining from beneficial intellectual, physical and social activities.

Thus, there is scientific evidence of a large variety of variables that can influence cognitive enrichment, such as biological, socio-economic, environmental factors, etc. There is a broad corpus of research literature supporting the importance of psycho-behavioral factors intervening in the ways of cognitive aging, specifically cognitive functioning, positive emotion and control, personality traits, psychosocial, physical activity and lifestyles [129]. The following briefly describes some of the factors considered most relevant: cognitive training and physical exercise.

#### Cognitive and Physical Training

First of all, education is the most consistent predictor of cognitive level and rate of change; even the plasticity brain mechanism can be modulated by education [158,159,160,161,162,163]. Education consistently predicts change in crystallized abilities and memory, and even with controlling factors such as age, gender, race and health, the effects of education on cognitive change are maintained. Although, in a recent longitudinal study of subsamples of older adults with and without dementia, higher educated individuals were seen to perform better at baseline; these performance benefits were nullified at 10-year follow-up [164].

The relationship between education and cognitive performance in older ages, as has mentioned previously, might be due to the possibilities of higher employment status, greater income, better health insurance or social and financial support. Thus, evidence regarding the influence of status and work complexity on cognition suggests that it is related to the maintenance of cognitive abilities at older ages [34,159,165,166,167]. Moreover, many older adults want to stay in the labor market after the official retirement age, thus slowing possible cognitive decline [167], while later retirement has also been associated with delaying the onset of AD [168]

Moreover, maintaining an intellectually stimulating lifestyle predicts better maintenance of cognitive skills, fewer memory issues and better daily functioning [169,170,171,172]. Even leisure activities and/or complex activities protect against cognitive decline in humans [173,174,175]. Some researchers have suggested that older people who engage in mentally stimulating activities may have had some advantages through their life span: a higher socioeconomic status that allows them to engage with more activities, having had a higher educational level and other variables with respect to the quality of health care. Consistent with this idea, it has been proved that higher incomes predict slower cognitive decline [141,176]. Furthermore, some studies have examined the relationship between early-life SES and cognitive decline in old age, and childhood SES has been associated with health status, health behaviors, major depression and physical functioning in old age, all of which are linked to a decline or maintenance of cognitive performance in later life [173,177,178,179].

Finally, with regard to cognitive training, developed under the hypothesis “use-it-or-lose-it” during recent decades, studies have focused on cognitive plasticity, which is operationalized as the extent to which an individual can improve his/her performance in a given cognitive task through training [24], based on evaluation and intervention studies with experimental methods (see Table 2). Although several systematic reviews have highlighted that cognitive interventions improved cognitive performance only in the domain trained but not in other domains (moderate-strength evidence) [180,181,182], and they are not generalized to everyday situations [183,184], other systematic reviews and meta-analyses have shown evidence for small but consistent effects of cognitive interventions in improving cognition in healthy populations of aging adults, and that the results can be generalized to other mental abilities on non-trained measures [180,185,186,187]; in addition, continued plasticity until age 80 and above is possible [150,188]. Along these lines, an analysis based on data from four major longitudinal studies in cognitive activity predicting cognitive outcomes over up to 21 years found that a change in cognitive engagement was associated with change in cognitive performance, although baseline activity at an earlier age and engagement did not predict rates of decline later in life, suggesting that change in cognitive activity from one’s previous level has at least a transitory association with cognitive performance measured at the same point in time [189]. Furthermore, Duda and Sweet’s review of cognitive training programs provide evidence of neural effects in the frontoparietal network [190].

Therefore, we can find interventions that have been designed to maintain cognitive performance in old age and have shown benefits in a variety of domains: memory performance [169,192] and global cognition; fewer memory issues [170,193]; changes such as an increase in the number of physical, cultural, intellectual and social activities carried out; improved lifestyles (diet and physical exercise); and greater self-efficacy for aging and life-satisfaction [194]. Although, it seems that without additional practice, memory performance tends to revert to the original level [195]. Jones et al. showed that reasoning training attenuated aging-related change, and persons trained in memory retained 125% of their initial training-related gains at approximately 5 years after training [196].

New technologies are present in all areas of our lives, and cognitive training is one of them. Electronic (e.g., computer and video game based) cognitive training requires few resources (home computer with internet access), so it is becoming more relevant and expands accessibility of training to a broader number of individuals. There has been a great increase of brain-training products (see Table 3), and although Rebok et al. have shown that computerized cognitive training in independent older adults improve cognitive and functional benefits, even 10 years later, few of them have demonstrated cognitive benefits, and “transfer effects”; that is, improvements being generalized to everyday cognition and daily functioning [191,197]. Some of these interventions have shown an improvement in participants’ well-being [198].

In summary, results from training studies have indicated that the majority of healthy older adults improve cognitive performance after cognitive training or practice (for a review see [133,191]), although not all of them have shown generalizability to the older person’s daily life.

Regular physical activity and exercise is one of most important lifestyle factors having a positive impact on successful aging as well influencing the cognitive health of older adults. Physical activity is defined by WHO as any bodily movement produced by skeletal muscles that requires energy expenditure. It has been suggested that “Fitness is serving a neuroprotective function for the aging human” [203].

**Figure 4:**
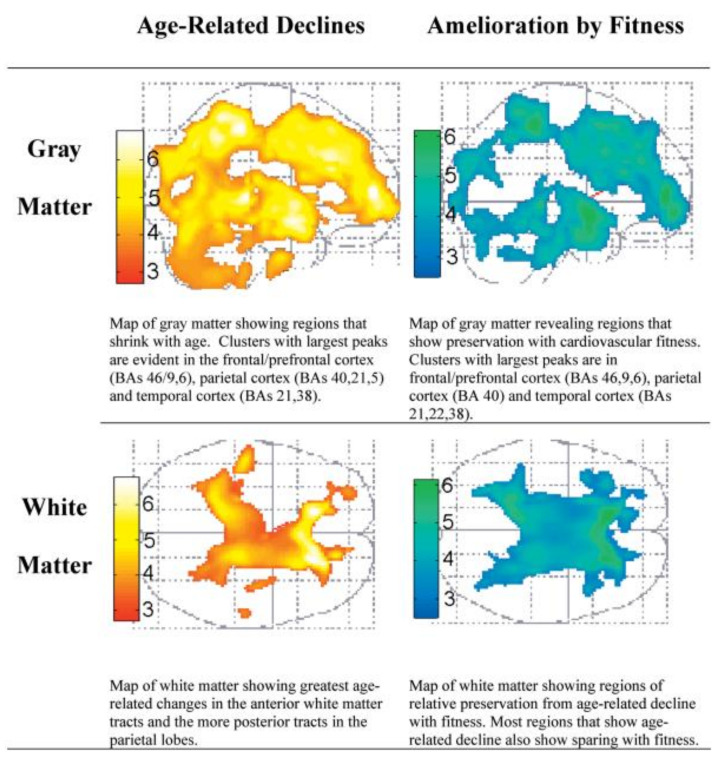
Statistical maps derived from multiple regressions of age and cardiovascular fitness on gray (**top row**) and white matter (**bottom row**) density [214]. The brighter colors represent greater tissue density changes with age (**left side**) and greater sparing of tissue density with increasing fitness (**right side**). Reproduced with permission from Oxford University Press, J Gerontol A Biol Sci Med Sci. 2003. License 4967121230914.

Cardiovascular exercise has been associated with improved cognitive functioning in aging humans [204,205]. Physical activity enhances older adults’ cognitive function and reduces the progression of age-related cognitive decline in healthy older adults [173,206,207,208,209,210]. It is even associated with increased hippocampal volume, and Colcombe et al. reported significant increases in brain volume, in both gray and white matter regions, as a function of fitness training for the older adults who participated in aerobic fitness training (see Figure 4) [211,212].

A relevant final study reviewing epidemiological (i.e., longitudinal cohort) and intervention studies on the role of physical activity and exercise in promoting cognitive health in older adults shows that it is associated with a 38% lower risk of cognitive decline, improves several aspects of cognition and reduces age-related changes in brain regions implicated in executive functions, learning and memory in older adults [213]. Furthermore, it predicts a 28% lower risk of developing any type of dementia and a 45% lower risk of developing Alzheimer Disease (AD) [213] (for a review see Table 4).

In sum, promoting regular cognitive training and physical exercising as healthy behavioral lifestyle options leads to healthy habits with a significant repercussion on cognitive functioning. There is an important corpus of empirical evidence regarding the association between regular cognitive training and physical exercise, showing that it must be complemented with other healthy habits such as healthy diet, no smoking and drinking moderately, coping with stress and having contact and support within a social network, to enrich cognitive functioning across older adulthood.

## 4. Conclusions

Life has lengthened. We reach more advanced ages, with the probability of reaching an old age in good health. There is a great heterogeneity between older adults, and positive aging is possible. As is well known and supported by both cross-sectional and longitudinal studies, older adults show a great range of inter-individual variability in cognitive functioning changes attributed to age. Authors furthermore agree that when decline occurs in some abilities, there is stability or even growth in others. Nevertheless, performance on cognitive tasks that involve processing speed, working memory and cognitive plasticity steadily declines after midlife, although rapid changes in cognitive abilities are usually signs of disease and appear unrelated to age. These inter-individual differences are not only explained by chronological age but variance is also shared between processing speed and the two facets of intelligence (fluid and crystallized abilities).

Intellectual functioning in healthy individuals seems to decline rather late in life, if ever. Longitudinal studies have shown that age-related decline in cognitive functioning occurs later in life than was indicated by cross-sectional studies. It seems that the majority of healthy individuals in their eighth decade preserve their cognitive abilities. Longitudinal comparisons may be distorted when considering the influence of time or period of measurement. Much more research is required regarding this aspect.

Experimental studies carried out in natural situations have shown that cognitive functioning can be optimized and/or compensated across healthy lifestyles by including regular cognitive training and physical exercise as well as a supportive environment, together providing for a healthy life. In sum, cognitive functioning and intellectual competences can be promoted, and intelligence can be trained. Engaging in intellectually and mentally stimulating activities shows lower rates of cognitive decline. There is also evidence demonstrating the benefits of aerobic physical exercise on cognitive functioning in older adults. Furthermore, exercise and environmental enrichment lead to cell proliferation in critical areas of the central nervous system.

## Figures and Tables

**Table 2:** Examples of experimental/program evaluation and meta-analyses studies of cognitive training for enhancing and promoting cognitive functioning.

Type of Information Presented	Study	Program Structure	Results Obtained
Experimental/evaluation studies	Longitudinal survivors of the Berlin Aging Study (*n* = 96) [184]	The training program comprised a total of eight 1–2 h sessions, scheduled 1 week apart of mnemonic practice. Training took place in individualized sessions at home.	85% of the 75 to 101-year-old participants were not able to improve their memory performance by any substantive amount during adaptive practice.
	Ten-year follow-up of a randomized, controlled single-blind trial with 3 intervention groups and a no-contact control group [191].	Ten-session training for memory, reasoning, or speed-of-processing.; 4-session booster training at 11 and at 35 months after training.	Results showed cognitive intervention resulted in less decline in self-reported IADL compared with the control group. Reasoning and speed, but not memory, training resulted in improved targeted cognitive abilities for 10 years.
Meta-analyses or systematic reviews	Meta-analysis of 6 randomized controlled trials of cognitive training interventions for healthy individuals (lasting at least 6 months; follow-up ranged from 6 months to 2 years, comparing cognitive training with usual care, waitlist, information, or attention controls in adults without dementia [180]	Trainings for healthy older adults were computer based or a combination of computer and noncomputer (paper-and-pencil) interventions	Training improves cognitive performance in the domain trained. Evidence was insufficient regarding whether cognitive training reduces the risk for future mild cognitive impairment (MCI) or dementia
	A Systematic Review and Meta-Analysis of 22 Randomized Controlled Trials in healthy y participants older than 60 years [186]	Working Memory Training. The total number of training sessions ranged from 3 to 25 (median = 10 sessions) and the total training hours ranged from 1.5 to 17.25 (median = 10 h). The training frequency ranged from 1.5 to 5 sessions per week. The pre- to follow-up interval ranged from 3 to 18 months	Results can be generalized to other mental abilities on non-trained measures, improving processing speed and reasoning in late adulthood.
	A Meta-Analysis of 49 studies, containing 61 different experiments or independent subject groups for older adults (range: 63–87 years) [73]	Executive control and working memory training with total number of training sessions ranged from 7.96 to 16.66 (median = 9.81 sessions) and the total training hours ranged from 8.24 to 10.69 (median = 8.93 h).	Results showed significant and large improvements in the trained tasks and in near-transfer measures (tasks not explicitly trained, but measuring the same construct as the construct trained)

**Table 3:** Examples of structured computer-based cognitive training.

Name of the Program	Program Structure	Results Obtained
Brain HQ by Posit Science (brain hq.com)	This program focuses on six categories: Attention, Memory, Brain Speed, Intelligence, People Skills, and Navigation. Ten-session training was conducted in small groups in ten 60–75 min for memory, reasoning, or speed-of-processing; 4-session booster training at 11 and at 35 months after training. Memory training focused on improving verbal episodic memory through instruction and practice in strategy use. Reasoning training focused on improving the ability to solve problems that contained a serial pattern. Speed-of-processing training focused on visual search and ability to process increasingly more complex information presented in successively shorter inspection times	It achieves immediate improvement in the trained cognitive ability (memory, processing speed and attention). These improvements dissipated slowly but persisted for at least 5 years for memory training and for 10 years for reasoning and speed-of-processing training, as well as less difficulty in performing IADL activities [191].
Cognifit	A program for 8–10 weeks with a total of 40 sessions, 1 h per day, of sensory and cognitively demanding exercises where, to make progress in tasks, the participant must perform increasingly more difficult stimulus recognition, discrimination, sequencing, and memory tasks under conditions of close attentional control, high reward, and novelty.	The authors found significant post-training improvements in healthy older adults, on untrained tests of attention, memory, executive functioning, visuospatial abilities and focused attention, although ecologically valid tasks of everyday cognitive functioning was not evaluated [199]
Cogmed QM (Pearson)	5 weeks of computerized training on various spatial and verbal working memory (WM) tasks using a commercial software product (Cogmed QM), which runs on the participants’ PCs at home. Individuals trained for 20–25 days (minimum 20 days) on seven verbal and non-verbal WM tasks. All tasks involved: (1) maintenance of multiple stimuli at the same time; (2) short delays during which the representation of stimuli should be held in WM; and (3) unique sequencing of stimuli order in each trail. Performance was assessed before training, after 5 weeks of intervention, as well as after a 3-month follow-up interval.	Significant improvements in trained and untrained neuropsychological tests of verbal and non-verbal working memory, sustained attention and working memory, as well as self-report of cognitive functioning at post-training and 3-month follow. Improvements were not seen in the areas of memory, nonverbal reasoning, or response inhibition. The generalizability of training to more ecologically valid everyday tasks was not assessed [200]
Nintendo DS Brain Training or Wii Big Brain Academy programs	Two studies were carried out. In the first, participants followed 5 days/week for four weeks, for a total of 20 h of Wii Big Brain Academy practice over the course of 1 month and, in a second month, completed 20 one-hour reading sessions with articles on 4 different current topics. The Nintendo DS Brain Training package consists of a series of games, or puzzles with: Math calculations, Verbally-based games, Working memory games and Mental rotation. In the second study, participants used Nintendo DS regularly over a 6-week period.	Modest improvements, but did not achieve improvements in untrained cognitive abilities [201,202].
Dakim Brain Fitness	A 6-week healthy lifestyle program consisted of 60-min classes held twice weekly. The educational program focused on memory training, physical activity, stress reduction, and healthy diet	This program showed improvement in delayed memory after 2 months and 6 months, but no in immediate memory or verbal abilities [171].

**Table 4:** Examples of experimental/program evaluation and meta-analyses studies of regular physical activity and exercise for enhancing and promoting cognitive functioning.

Type of Information Presented	Study	Program Structure	Results Obtained
Experimental studies	An intervention study with a control group of a volunteer sample of 55–70 year old sedentary individuals [215].	An aerobic training program in strength and flexibility exercises. The exercise groups met in three one-hour sessions a week over a four-month period.	The aerobic training group showed improved cardiorespiratory function, and a significantly greater improvement on the neuropsychological test battery than did either control group.
	A randomized intervention study with 10 healthy men and 30 healthy women, ranging in age between 63 and 82 years [216].	A 10-week aquatic fitness program. The aquatic exercise program consisted of three 45-min sessions per week.	Results showed a greater improvement in task conditions and switching abilities compared to conditions that do not require executive or attentional control processes.
	A randomized clinical trial with 57 older adults (65−79 years) [217].	A 10-month training program (aerobic versus strength and flexibility). Neurocognitive tasks were selected to reflect a range from little (e.g., simple reaction time) to substantial (i.e., Stroop Word–Color conflict) executive control.	The positive effect on executive control was observed after aerobic training only.
	Randomized controlled trial, 70 healthy senior citizens (age 60–75) [218]	Combined training group (physical and cognitive) The interventions took place in groups of 8–10 participants *Physical Activity Intervention*: moderate aerobic endurance training combined with moderate strength training. Participants trained two times per week, each session lasting 60 min, for a period of 16 weeks *Cognitive Activity Intervention*: once a week for approximately 30 min. *Combined Physical Plus Cognitive Activity Intervention*: the physical plus cognitive interventions, twice a week. The cognitive training program was carried out at the first training session of the week, before the physical training. The total duration of the first training session each week therefore was 90 min, while the second session lasted only 60 min (consisting only of physical training). *Waiting Control Group*	The physical, cognitive, and combined training groups enhanced their concentration immediately after intervention. Only the physical training group showed improved concentration 3 months later. The combined training group displayed improved cognitive speed both immediately and three months after intervention. The cognitive training group displayed improved cognitive speed 3 months after intervention.
Meta-analysis	A meta-analysis of 18 intervention studies with control groups [204].	A diverse of aerobic fitness training, which could be divided into two groups: those that emphasized cardiovascular fitness in isolation (aerobic) and those that combined cardiovascular fitness training with strength training (combination). The training session could vary from 15–30 min; 31–45 min; and long, 46–60 min. And the interventions could last from 1–3 months; 4–6 months; and 6 months.	The results showed robust but selective benefits for cognition, with the largest fitness-induced benefits occurring for executive-control processes.

## Supplementary Materials

The following are available online at https://www.mdpi.com/1660-4601/18/3/962/s1, Table S1: A summary of selected studies on cognitive change in healthy older adults.
