# Supplementary material for: Cognition in Healthy Aging
Source: Int J Environ Res Public Health. 2021 Jan 22;18(3):962. doi: 10.3390/ijerph18030962 (PMC7908458; doi:10.3390/ijerph18030962)
Supplement: Supplementary file 1 [file ijerph-18-00962-s001.pdf]

Supplementary Table S1.
Table S1: A summary of selected studies on cognitive change in healthy older adults.

Study	Sample	Design	Measurement	Main Conclusions
Schaie (1983).	N= 2,193 participants followed over 7 years, 1,189 over 14 years, 662 over 21 years	Longitudinal and cross-sectional	Measures of Verbal Meaning, Space, Reasoning, Number and Word Fluency	There is no uniform pattern of age-related changes across all intellectual abilities, fluid abilities tend to decline earlier than crystallized abilities.<br>Women, may decline earlier on fluid abilities, while men do so on the crystallized abilities. Moreover, while fluid abilities begin to decline earlier, crystallized abilities show steeper decrement once the late seventies.
Salthouse (1998).	- children (age 5-17, n = 3,155),<br>-college (age 18-22, n = 735) and non-college student adults (age 18-94, n = 1,580).	cross-sectional	Reasoning. Spatial visualization. Vocabulary. Verbal memory. Perceptual speed	The results indicated that the majority of age-related differences appear to be shared across different cognitive variables and are well predicted by individual differences in higher order factors<br>The independent age-related effects, after considering the relations of age to what all variables had in common, were small relative to the total age-related effects.
Finkel et al (1998)	N=85 individuals aged 41- 84 at first assessment.<br>From the Swedish Adoption/Twin Study of Aging	Longitudinal: 3 times of measurement separated by 3-year intervals	Verbal ability, spatial abilities, Memory tests, perceptual speed	Stable mean performance in the younger cohorts and longitudinal decreases in mean performance in the older cohorts.<br>Cross-sectional comparisons were more likely to indicate that older cohorts have equal or less variability than younger cohorts.<br>Heritability of the general cognitive ability factor showed significant longitudinal decreases over time in the older cohorts.
Smith and Baltes (1999).	N= 516 persons, aged 70-103.	Cross-sectional. wave of the Berlin Aging Study (BASE; N = 516). may 1990-june 1993	The five ability factors are (a) Mental Mapping (perceptual speed), (b) Memory, (c) Reasoning, (d) Verbal Knowledge, and (e) Verbal Fluency. The first three abilities are indicators of fluid intelligence (gf), and the last two define crystallized intelligence (gc).	Significantly higher negative age correlations for the 3 mechanic abilities (perceptual speed; memory; reasoning) than the 2 pragmatic abilities (knowledge; fluency) up to the age of 70. The differences between the 2 dimensions of intelligence appear to decrease with increasing age
Linderberger y Reischies (1999).	N= 516 persons aged 70-103.	Cross-sectional	The BASE cognitive test battery. In this report they focused on 3 measures: 1) Enhanced Cued recall test, to estimate interindividual differences in encoding specificity. 2) The MMSE and 3) an index of brain atrophy base don a CT scan of the brain	The distinction between fluid and crystallized intelectual abilities extends into old and very old age, but compared with earlier periods of the life-span, this distintion appears to be less pronounced.<br>About a third of the interindividual variability was related to chronological age; the highest relationship was found for perceptual speed (38% if the reliable variance).
Caskie, Schaie y Willis (1999).	N=1.745 participants from 11 cohorts of the Seattle Longitudinal Study (SLS). Aged 25 to 81	cohort-sequential study design	The Verbal Meaning, Spatial Orientation, and Reasoning sub-tests from the Thurstone Primary Mental Abilities Test<br>Verbal, spatial, and reasoning ability raw scores were re-scaled to a T-score metric to allow all abilities to be compared on the same measurement scale	Rate of decline over time in all abilities was related to level of ability at age 67, the age at which decline tends to begin.<br>Cohort, gender, and level of education explained individual variation in the rate of decline for spatial ability. The rate of decline in verbal ability was predicted by cohort, and rate of decline in reasoning ability was predicted by both cohort and education<br>Being in a later birth cohort and having a higher level of education were both associated with higher levels of ability at age 67. Women had significantly higher levels of verbal and reasoning ability at age 67 than men, but men had significantly higher spatial ability at age 67 than women.

Study	Sample	Design	Measurement	Main Conclusions
Hassing et al (2002)	N=466, aged: 80-98 From the ongoing population- based longitudinal study Origins of Variance in the Old-Old (OCTO-Twin Study	longitudinal: 3 examinations at 2-year intervals.	Eleven tests that represent crystallized knowledge and five abilities associated with fluid abilities, and speed.	In crystallized knowledge and episodic memory, individuals with a shorter survival (deceased between T2 and T3) showed a steeper decline compared with those who survived longer.<br>Level of cognitive performance in late life is associated with proximity to death, that this relationship is longstanding, and that it is partially influenced by compromised cardio- and cerebrovascular functioning.
Deborah Finkel & Nancy L. Pedersen (2004)	Q1 (1984), N=2.018, over 50=548 pairs<br>Q2 (1987), N=1.637, over 50=420 pairs<br>Q3 (1990) N=1.496, over 50=403 pairs<br>Q4 (1993) N=1.450, over 50=385 pairs<br>Q5 (2003) N=1.309<br>Aged: 50-85yeasr old	Longitudinal and cross-sectional. 3-year interval between measurement occasions (in IPT5 the testing interval was 7 years).	In-person testing (IPT) involved an interview, administration of cognitive tests and a health examination of a subset of the SATSA twins.<br>The cognitive battery was designed to represent the domains of crystallized and fluid intelligence, and memory.<br>Additional measures of spatial ability and perceptual speed	Stability or even improvement for measures of crystallized ability up to age 70, followed by significant decline. Cognitives tasks with a large speed component show a significant acceleration in linear decline after age 65.<br>Declines in heritability in late adulthood with associated increases in environmental variance. The genetic variance in cognitive functioning in the middle-aged cohort was defined primarily by motor speed, whereas genetic variance in the older cohort was defined by perceptual speed.
Schaie, Willis y Caskie, 2004.	N=9,476 complete records on 4,857 participants. 2,193 participants followed over 7 years,	Longitudinal	Measures of Verbal Meaning, Space, Reasoning, Number and Word Fluency	Fluid abilities tend to decline earlier crystallized abilities. Women, may decline earlier on fluid abilities, while men do so on the crystallized abilities.<br>Results from the SLS have conclusively demonstrated the prevalence of substantial generational (cohort) differences in psychometric abilities<br>The variables most intensively studied thus far that have been implicated in reducing risk of cognitive decline in old age have included: (a) Absence of cardiovascular and other chronic diseases ; (b) favorable environment mediated by high SES ; (c) involvement in a complex and intellectually stimulating environment ; (d) flexible personality style at midlife ; (e) high cognitive status of spouse ; and (f) maintenance of high levels of perceptual processing speed.<br>Observed decline in many community dwelling older people is likely to be a function of disuse and is therefore reversible for many. Training effects are long-lasting with the trained participants still at an advantage over their controls after 7 and 14 years
Li, S.-C., Lindenberger, U., Hommel, B., Aschersleben, G., Prinz, W. & Baltes, P.B. (2004)	N=356, aged 6- 88 years old, uniformly distributed across 31 age bins	cross-sectional	15 psychometric tests from the Berlin Aging Study. The five ability factors are (a) Mental; (b) Memory, (c) Reasoning (d) Verbal Knowledge, (e) Verbal Fluency,. The first three abilities are indicators of fluid intelligence (gf), and the last two define crystallized intelligence (gc).	Maximum processing speed, processing robustness, and fluid intelligence were achieved by individuals in their mid 20s. Decrements were already visible by the mid 30s While the maximum crystallized intelligence scores were achieved by individuals in their 40s, and crystallized intelligence scores remained relatively stable until old age, at which point they also declined (beyond 70 years of age).<br>A larger percentage of the predicted variance in chronological age was jointly shared between processing speed and the two facets of intelligence, amounting to 69% of the explained variance in old age.
Tucker-rob y Salthouse (2008).	N = 2,227<br>24-91years<br>The dataset was aggregated from seven different studies conducted since 2001 at the Cognitive Aging Lab at the University of Virginia	cross-sectional	a battery of between 14 and 16 cognitive tests (3 or 4 for each ability) selected to measure fluid reasoning (Gf), spatial reasoning (Gv), verbal knowledge (Gc), processing speed (Gs), and episodic memory (Gm).<br><br>For the current study, all variables were standardized to the IQ metric (M = 100, SD= 15) for the youngest age	Contrary to the dedifferentiation hypothesis, there was no evidence for systematic increases in the magnitudes of relations among cognitive abilities.

Study	Sample	Design	Measurement	Main Conclusions
	Participants were divided into seven approximate 10-year age groups		group.	
Finkel et al (2009)	N=462<br>Aged: 50-91 years old	Longitudinal, 3-year intervals.	Wechsler: memory, visuoespatial, verbal, processing.	Individuals with more complex work demonstrated higher mean performance on the verbal, spatial, and speed factors.
Muniz et al (2009).	N=2.053 at baseline N=304 in the 4th interview<br>Aged:75+	Prospective cohort study. After baseline, interviews were conducted on all the survivors an average of 2, 7 and 9 years later.	MMSE	Higher educational level and social class, better mobility and younger age at baseline were identified as statistically significant positive factors for cognitive performance. Women and participants with better mobility were found to experience a slower decline with age than men and participants with poorer mobility.<br>Higher levels of education do not appear to protect against cognitive decline, though if the MMSE is used in the diagnostic process, individuals with less education may be diagnosed as having dementia somewhat earlier
Brown, MT (2010).	N=16.513 subjects and 53,900 observations. Aged 65+<br>From the Asset and Health Dynamics among the Oldest-Old study (AHEAD) and the HRS.	Longitudinal	Modified version of the TICS instrument.	A combined history of childhood disadvantage and psychiatric problems more strongly affects cognitive function, but cognitive declines remain consistent with those associated with psychiatric history. These effects are partially mediated by later-life demographic, socioeconomic, or health characteristics.
Gerstorf et al (2011).	N=6000, aged: 50-101<br>Subsample from the SLS who (a) were born between 1886 and 1913 (earlier born cohort) or 1914 and 1948 (later born cohort)	Cohort study.	5 primary mental abilities from the Seattle Longitudinal Study	The later born cohort showed steeper mortality-related declines.<br>Average levels of abilities at 3 years prior to death were up to one standard deviation below the mean of the crosssectional (at study entry) sample
Salthouse (2011).	N=1,576 adults, aged 19 to 95 years old	Longitudinal: 2 times<br>The range of test-retest intervals was 0.8 to 8.4 years, with a mean of 2.5 and a standard deviation of 1.1.	Four different abilities: reasoning ability; spatial visualization ability; episodic memory ability; and perceptual speed ability	The results suggest that the relations between age and cognitive change over intervals of less than 8 years are largely influenced by factors operating at or near the initial test occasion.<br>Increased age has weak, and possibly nonexistent, relations with the time-dependent component of cognitive change.
Fisker et al (2013)	N= 590, aged 44 to 88 years at first measurement.	Longitudinal: follow-up occasions at intervals of 3 years.	Crystallized abilities and fluid abilities. Memory. Finally, perceptual speed	Stability for measures of crystallized ability, linear age changes for many cognitive abilities, and a significant acceleration in linear decline after age 65 for measures with a large speed component.<br>Gender differences were found only in mean level, not in rate of decline.
Salthouse (2013).	Aged: 18-97 years old<br>2001: N=205; 2002: N= 269<br>2003: N= 330; 2004: N=458<br>2005: N=670;2006: N=913<br>2007: N=444;2008: N=255<br>2009: N=236; 2011: N=440	Cohort Study	Reasoning; Spatial visualization; Vocabulary; verbal memory; Perceptual speed.	Within-cohort differences across ages were often as large as between-cohort differences across ages.
Yam et al (2014).	N=698, aged 65–94, from the no-treatment control group of the ACTIVE studio	Longitudinal. (N=698) and 1 (n=582), 2 (n=551), 3 (n=511), 5 (n=452), and 10 (n=249) years following the baseline occasion.	The ACTIVE study's cognitive battery was comprised mostly of age-sensitive measures of information processing and fluid intelligence (Horn & Cattell, 1967; Baltes et al., 1980). Four areas of cognitive ability were assessed within the battery. Inductive reasoning, Episodic	Modeling revealed an overall inverted-U shape (quadratic) trajectory across cognitive domains. Level and slope in reasoning demonstrated the closest association to level and slope of everyday cognition, and accounted for most of the individual differences in linear gain in everyday cognition.

Study	Sample	Design	Measurement	Main Conclusions
			verbal memory, Visual processing speed, vocabulary, Everyday cognition.	All cognitive variables demonstrated a negative quadratic (inverted-U shape) trajectory, indexing decline in cognition over time. The most pronounced quadratic slope was observed for speed
Salthouse y Soubelet (2014).	**Study 1:** N=2.250, aged 65+, from the Virginia Cognitive Aging Project (VCAP)<br>**Study 2**: N= n 4,775<br>Aged: 50.8 (18.0)	Longitudinal and Cross-sectional	12 neuropsychological tests selected to reflect four different abilities: reasoning ability; spatial visualization ability; episodic memory ability; and perceptual speed ability	High values of within-person variability may have specificity in predicting late life cognitive decline.
Hatta et al (2015).	N=338 healthy rural community dwellers, aged: 50+	cross-sectional design	The Nagoya University Cognitive Assessment Battery (NU-CAB)	There was a sex difference in the cognitive-decline trajectories for verbal and visuospatial task performance after the 50s. Women showed better performance than men in the age group of 60s, while men showed better performance than women in the age group of 80s. For women, performance of both verbal and spatial tasks declines gradually as age increase, but remain stable from 50s to 60s and then declines linearly from 60s to 80s. For men, performance of both verbal and spatial tasks declines gradually as age increases and there was no turning point of prominent cognitive decline.
Downer et al (2016).	N=1.336 individuals<br>Aged: 75+	Longitudinal. During four Waves from 2004–2005 to 2012–2013.	MMSE	Nearly 31% of the final sample maintained high global cognition (persistent high), 52.6% experienced slight decline (decline but high), and 15% experienced severe decline in global cognition (decline to low).<br>Advancing age, requiring help with one or more ADLs, and high depressive symptoms were all significantly associated with being in the decline to low trajectory class, whereas greater educational attainment was significantly associated with increased likelihood in the decline but high and persistent high trajectory classes. Participants who were not married and lived alone were 2.16 times as likely as those who were married and living with another person to be in the decline but high trajectory class.
Salthouse (2016).	cross-sectional N=5,014)<br><br>longitudinal N =1,353, a subset from the cross-sectional sample<br><br>Aged: 18-99y	cross-sectional and 3-occasion longitudinal	Reasoning. Spatial visualization. Vocabulary. Verbal memory. Perceptual	Adult age appears to have weak relations with specific measures of cognitive functioning, defined as independent of influences shared across different types of cognitive measures, and that this is true in both crosssectional and longitudinal comparisons.<br>The longitudinal age relations differed from the cross-sectional relations in exhibiting increases rather than decreases in the average score among adults younger than about 50 years of age.
Salhouse (2017).	N= 2.546 adults<br>Aged: 18- 95y	Longitudinal: 2 ocassions	Reasoning. Spatial visualization. Vocabulary. Verbal memory. Perceptual speed	Although increased age was associated with specific influences on speed in cross-sectional comparisons, and in memory change in longitudinal comparisons among older adults, most of the relations between age and cognitive functioning in both cross-sectional and longitudinal comparisons were manifested as general influences shared with other cognitive measures.
